# Tetra-color superresolution microscopy based on excitation spectral demixing

**DOI:** 10.1038/s41377-022-01054-6

**Published:** 2023-01-02

**Authors:** Wanyan Wu, Shihang Luo, Chunyan Fan, Tianjie Yang, Shuwen Zhang, Wenxiang Meng, Tao Xu, Wei Ji, Lusheng Gu

**Affiliations:** 1 Institute of Biophysics, Chinese Academy of Sciences, Beijing, 100101 China; 2 College of Life Science, University of Chinese Academy of Sciences, Beijing, 100049 China; 3 State Key Laboratory of Molecular Developmental Biology, Institute of Genetics and Developmental Biology, Innovation Academy for Seed Design, Chinese Academy of Sciences, Beijing, 100101 China; 4 School of Future Technology, University of Chinese Academy of Sciences, Beijing, 100049 China; 5 College of Advanced Agricultural Sciences, University of Chinese Academy of Sciences, Beijing, 100049 China; 6 Bioland Laboratory, Guangzhou, 510005 China; 7 Guangzhou Laboratory, Guangzhou, 510030 China

**Keywords:** Super-resolution microscopy, Total internal reflection microscopy

## Abstract

Multicolor imaging allows protein colocalizations and organelle interactions to be studied in biological research, which is especially important for single-molecule localization microscopy (SMLM). Here, we propose a multicolor method called excitation-resolved stochastic optical reconstruction microscopy (ExR-STORM). The method, which is based on the excitation spectrum of fluorescent dyes, successfully separated four spectrally very close far-red organic fluorophores utilizing three excitation lasers with cross-talk of less than 3%. Dyes that are only 5 nm apart in the emission spectrum were resolved, resulting in negligible chromatic aberrations. This method was extended to three-dimensional (3D) imaging by combining the astigmatic method, providing a powerful tool for resolving 3D morphologies at the nanoscale.

## Abstract (web summary)

A fast switching scheme-based excitation resolving method was proposed for fluorescent molecule identification, which enables tetra-color SMLM imaging

## Graphical abstract

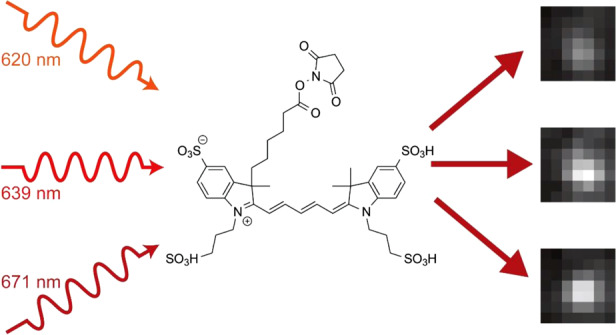

## Introduction

The development of single-molecule localization microscopy (SMLM)^1–3^ has provided a method for exploring nanostructures in cells that were previously concealed by diffraction limits, improving the spatial resolution of fluorescence microscopy to tens of nanometers^4^. In addition to the spatial resolution, multicolor imaging in SMLM is critical for investigating protein colocalizations and organelle interactions in biological research^5–7^.

The multicolor capability of SMLM is realized by single-molecule identification with intrinsic properties of different fluorescent dyes, such as fluorescence lifetime^8^, emission spectrum, and excitation spectrum.

Previous work has mainly focused on the emission spectra of fluorescent dyes. One approach used spectrally well-separated fluorophores^9^, requiring additional error-prone alignment procedures between channels^10,11^. Another approach used ratiometric detection to distinguish the fluorescence intensity in several channels^12–16^. A dispersive prism was also used to determine the spectral peaks of different dyes^17,18^, meanwhile, a dual-objective design was adopted because both locating single molecules and distinguishing spectra require a considerable number of photons.

Excitation spectra of fluorescent dyes can be also adopted in multicolor imaging. Exciting with different lasers has been proved in triple-color imaging of live cell^19^. Additionally, excitation spectral demixing has been applied in wide-field imaging^20^, and lasers with different excitation frequencies have been utilized for differentiating dyes in multicolor imaging^21^. However, these sequential imaging-based methods are not fully compatible with STORM imaging, due to the highly dynamic properties of single-molecule dyes.

Here we proposed a single molecule identification method that used the excitation spectrum of fluorescent dyes and was fully compatible with STORM imaging, called excitation-resolved stochastic optical reconstruction microscopy (ExR-STORM). This method is based on the fact that the fluorescence intensity is proportional to the excitation cross-section at each wavelength for a given laser intensity^21^. Using this method, we successfully resolved four spectrally very close far-red dyes simultaneously with negligible cross-talk and little chromatic aberrations by utilizing three excitation lasers with a single objective. A 4 kHz resonant mirror was adopted to enable fast switching among excitation channels; thus, three subimages could be obtained under different excitation wavelengths simultaneously within one exposure time^22,23^, enabling the identification of dyes in the realm of SMLM. ExR-STORM fully utilized photons in three light paths to locate and identify dyes through a co-fitting method, realizing high localization precision. Furthermore, by combining ExR-STORM with the astigmatic method^24^, we demonstrated the capability of 3D tetra-color imaging.

## Results

### Principle of ExR-STORM

**Fig. 1:**
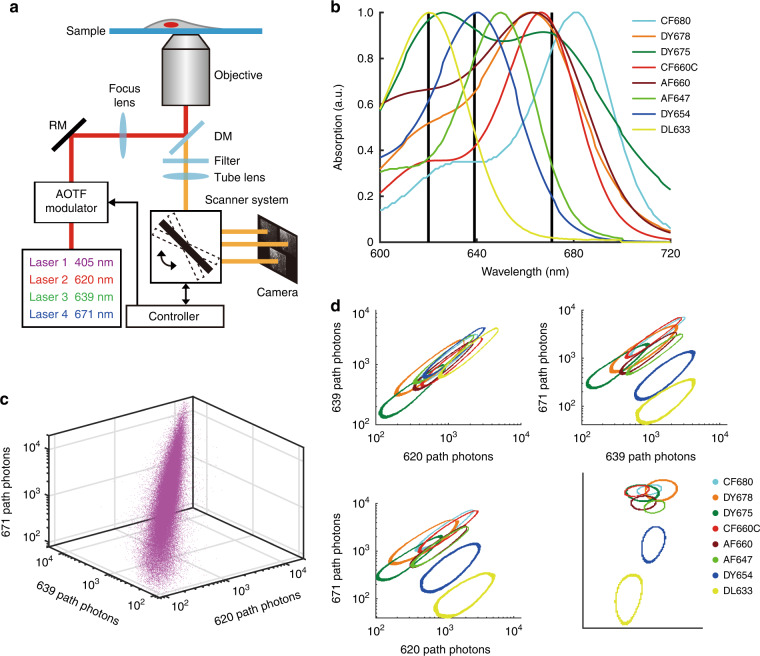
Working principle of ExR-STORM. **a** Scheme of the optical setup. Three excitation lasers (620, 639, and 671 nm) were combined and then modulated by an AOTF prior to the illumination of the sample. The laser switching was synchronized with the scanner, resulting in three subregions with different excitation wavelengths within one exposure time. **b** Absorption spectra of the eight fluorescent dyes. The three black lines correspond to the three excitation wavelengths. **c** Scatter plot of the photon number of Alexa Fluor 647 in the three excitation channels. **d** 2D projections of the photon number spatial occupation along the 671 path, 620 path, 639 path, and the isometric projection (which is an axonometric projection in which the three coordinate axes appear equally foreshortened and the angle between any two of them is 120°) for the investigated dyes, where the half-maximum contour of the distribution was selected as the boundary of the investigated dye; more than one million single molecules of each dye were selected for a statistical examination of the molecular behaviors

Most STORM dyes that exhibit superior performance are located in the far-red region^25^. In ExR-STORM, excitation lasers were selected for the candidate dyes, which could be differentiated based on the corresponding intensities under each laser. The optical setup scheme is shown in Fig. 1a. Four lasers (620 nm, 639 nm, 671 nm for excitation, and 405 nm for activation) were combined and modulated with an acousto-optic tunable filter (AOTF) before being focused on the back focal plane of the objective for illumination. In the imaging path, the fluorescence signals were focused on the surface of a 4 kHz resonant mirror, then sequentially reflected and refocused onto three subregions of the electron-multiplying charge-coupled device (EMCCD) with three pairs of lenses (Supplementary Fig. S1). The switching between subregions was synchronized with the AOTF to ensure that for each subregion, only the fluorescence signal excited by the corresponding laser was accumulated. The scanning cycle was 250 μs with a duty cycle of 84%, allowing brightness fluctuations caused by on-off state transitions and other effects to be minimized^22,23,26^ (Supplementary Fig. S2). With this method, three subimages of the same single molecule could be acquired under different excitation wavelengths within one exposure time (Supplementary Fig. S3). The molecule was then identified according to the intensities in the three subimages. For dyes in the multicolor sample, each kind of molecule can be recorded and identified simultaneously under the condition that the fluorophores are sparsely distributed in space to avoid overlapping.

ExR-STORM identified dyes based on the fact that the fluorescence intensity is proportional to the absorption cross-section at each excitation wavelength^21^. In this work, candidate dyes were selected based on their absorption spectra (Fig. 1b) and evaluated by our system. For every single molecule, 2D Gaussian fitting was performed to extract the location and photon numbers in three subimages. A 3D scatter plot of the photon numbers demonstrated a narrow cluster distribution (Fig. 1c), indicating that the molecules possessed highly consistent fluorescence intensity properties under the three excitation wavelengths. Next, we calculated the photon number distribution for each dye, with the half-maximum contour of the distribution selected as the boundary of the investigated dye (Fig. 1d). We found that each dye exhibited a distinct cluster distribution of photon numbers under the three excitation wavelengths. Thus, multicolor STORM imaging could be conducted when the proper dyes were selected. Because this demixing method does not depend on the emission spectrum, dyes with identical emission spectra could be demixed as long as they possessed distinguishable excitation characteristics. Using this method, chromatic aberrations can be minimized, and the choice of dyes for multicolor imaging is enriched.

### Demixing through ExR-STORM

**Fig. 2:**
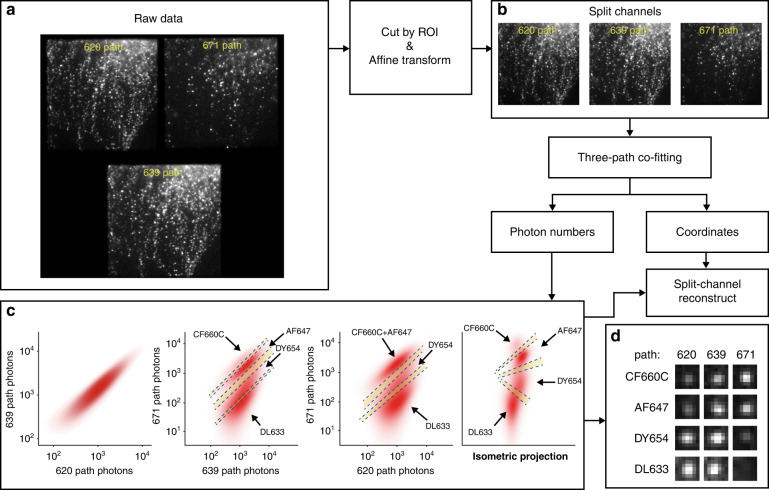
Workflow of ExR-STORM. **a** One frame of the raw image stack. **b** Split channels of the raw image based on the ROI of the three light paths, and transformed by the affine matrices to the 639 nm light path. **c** Diagram of dye assignment along the projections of the 671 path, 620 path, 639 path, and the isometric projection (which is an axonometric projection in which the three coordinate axes appear equally foreshortened and the angle between any two of them is 120 degrees), with the yellow regions between the dashed lines, rejected. **d** Single-molecule of each dye in the three light paths from (**b**) based on the classification process shown in (**c**)

The workflow of ExR-STORM is shown in Fig. 2. To illustrate our scheme, we selected DyLight 633, Alexa Fluor 647, Dyomics 654, and CF660C for tetra-color imaging. The recorded raw data (Fig. 2a and Supplementary Video 1) were first split and transformed to create 3 aligned subimage stacks (Fig. 2b). Following single-molecule detection and image extraction similar to the conventional STORM imaging process, the 3-channel images of every single molecule were co-fitted as modified 2D Gaussian models:

1 $$\left\{ {\begin{array}{*{20}{c}} {{\rm{PSF}}_1\left( {x,y} \right) = A_1{\rm{exp}}\left( { - \left( {\frac{{\left( {x - x_0} \right)^2}}{{2\sigma _x^2}} + \frac{{\left( {y - y_0} \right)^2}}{{2\sigma _y^2}}} \right)} \right) + B_1} \\ {{\rm{PSF}}_2\left( {x,y} \right) = A_2{\rm{exp}}\left( { - \left( {\frac{{\left( {x - x_0} \right)^2}}{{2\sigma _x^2}} + \frac{{\left( {y - y_0} \right)^2}}{{2\sigma _y^2}}} \right)} \right) + B_2} \\ {{\rm{PSF}}_3\left( {x,y} \right) = A_3{\rm{exp}}\left( { - \left( {\frac{{\left( {x - x_0} \right)^2}}{{2\sigma _x^2}} + \frac{{\left( {y - y_0} \right)^2}}{{2\sigma _y^2}}} \right)} \right) + B_3} \end{array}} \right.$$

where *A*_*i*_ and *B*_*i*_ denote the amplitude intensity and the background of single molecules in subimage stack *i* (*i* = 1, 2, 3, which correspond to the 620 path, 639 path, and 671 path, respectively), respectively; *σ*_*x*_ and *σ*_*y*_ denote the standard deviation in the X and Y directions, respectively; and *x*_0_ and *y*_0_ denote the position of a single molecule. The above parameters were extracted by our fitting model (1). The photon numbers in each light path can be calculated as:

2 $$N_i = \frac{{2\pi A_i\sigma _x\sigma _y\cdot {\rm{ADU}}}}{G}$$

where *N*_*i*_ is the photon number in the subimage stack *i*, ADU is the analog-to-digital unit conversion factor of the EMCCD, and *G* is the gain of the EMCCD.

For molecule identification, we first plotted the photon numbers of every single molecule in the three light paths in a 3D logarithmic coordinate system, each with a point (*N*_1_, *N*_2_, *N*_3_). Then, molecule assignment was conducted in the projections of the 671, 620, and 639 paths, as well as the isometric projection (Fig. 2c). It should be noted that the classification result of each dye was the intersection of the selected regions in four projections; otherwise, the molecule was rejected. After molecule identification, multicolor superresolution images were reconstructed according to the classification results and positions obtained previously. Typical single-molecule images of each dye in the three channels are shown in Fig. 2d.

**Fig. 3:**
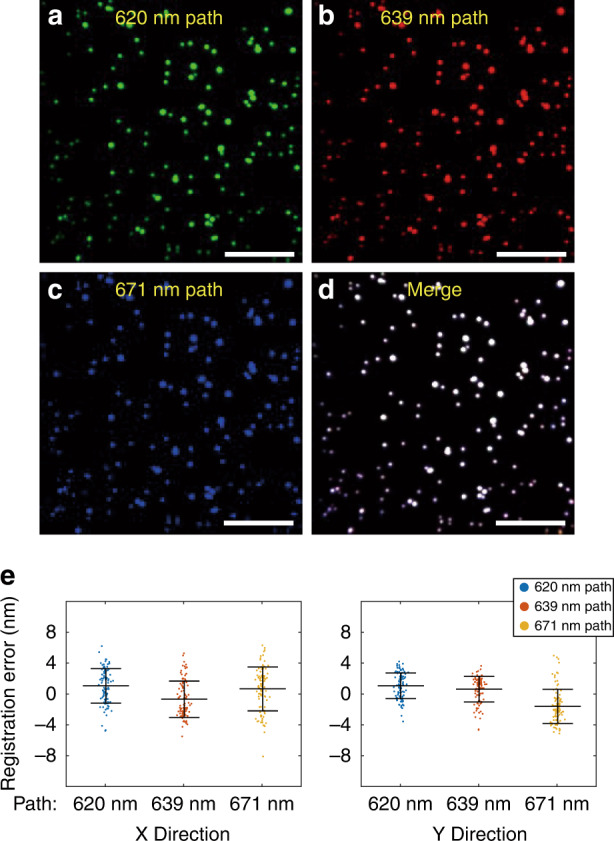
Image registration and verification of co-fitting method. 100 nm fluorescent microspheres were used for image registration and verification. **a**–**d** Transformed images of the three light paths and merged image. **e** Positions of independent fitting in each transformed channel relative to the co-fitting results in the X and Y directions. Error bars are presented as the mean ± standard deviation. Scale bars: 10 μm (**a**–**d**)

The co-fitting method was validated by microsphere images, as shown in Fig. 3. Three transformed subimages and the merged image are shown in Fig. 3a–d. The localization results obtained through independent fitting in each channel were compared with the co-fitting results, and the localization error and bias of each channel were analyzed (see Materials and methods). For the localization difference between independent fitting and co-fitting, the maximum mean bias and standard deviations were 2 and 3 nm, respectively (Fig. 3e), which were well below the localization precision of the ExR-STORM system (Supplementary Fig. S4).

### Tetra-color performance of ExR-STORM

The ExR-STORM identification process was conducted in three dimensions (*N*_1_, *N*_2_, and *N*_3_). Compared with the spectral demixing method^12–14,16^ that used a two-dimensional photon number distribution for dye classification, one additional dimension was used in our method. During our assignment process, four projections of the photon number distribution were selected, and the final classification was the intersection of the classifications in each projection, thus contributing to a more accurate classification result.

**Fig. 4:**
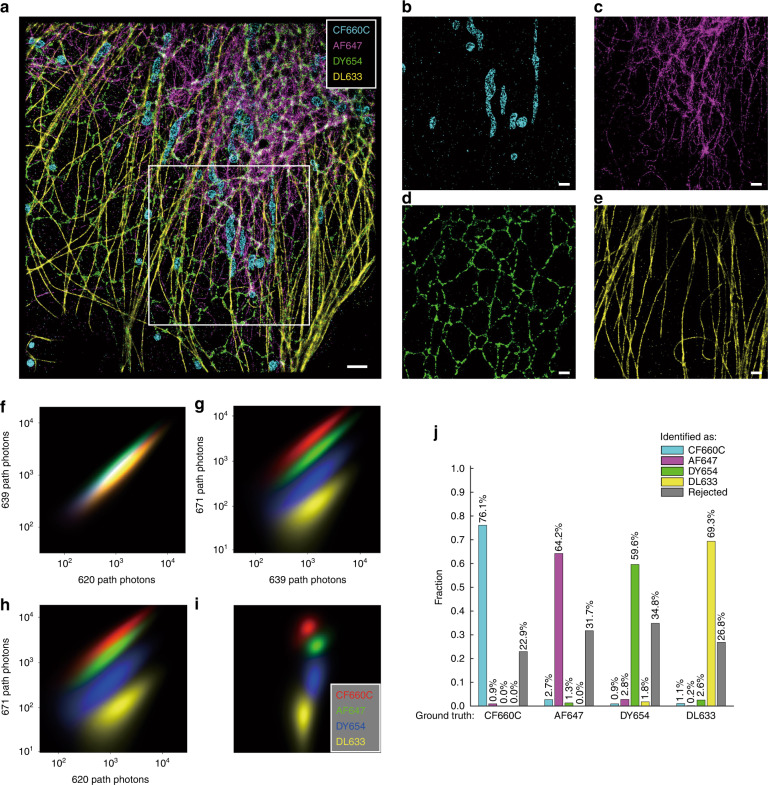
Tetra-color imaging based on ExR-STORM. CF660C, Alexa Fluor 647, Dyomics 654, and DyLight 633 were selected to label the outer mitochondrial membrane (cyan), intermediate filaments (magenta), endoplasmic reticulum (green), and microtubules (yellow), respectively, in a fixed COS-7 cell. **a** Four-color superresolution reconstruction image in which each detected single molecule was classified based on the excitation spectrum of the fluorophores. **b-e** Split channels of the white box region in (**a**). **f**–**i** 2D projections of the photon number distributions along the 671 path, 620 path, 639 path, and the isometric projection (which is an axonometric projection in which the three coordinate axes appear equally foreshortened and the angle between any two of them is 120°). **j** Cross-talk and rejected fraction for the four dyes. Scale bars: 2 μm (**a**); 1 μm (**b-e**)

This innovation enabled tetra-color imaging in fixed COS-7 cells. DyLight 633, Alexa Fluor 647, Dyomics 654, and CF660C were selected to label microtubules, intermediate filaments, endoplasmic reticulum (ER), and the outer mitochondrial membrane, respectively. With ExR-STORM, the four dyes were well resolved, and tetra-color superresolution images were successfully reconstructed (Fig. 4). The images of each channel were consistent with images of the separately acquired structures, and no obvious misidentification or cross-talk was observed (Fig. 4a–e).

By imaging four dyes in the cell sample individually, the cross-talk can be evaluated. The photon number distribution of each dye is plotted in Fig. 4f–i, and each distribution contains more than 1 million points. The classification was performed for each dye as previously described, showing a cross-talk of <3% and a rejection fraction of <35% (Fig. 4j and Supplementary Table 1). Take CF660C as an example, 76.1%, 0.9%, 0.0%, and 0.0% of the total single molecule events were identified as CF660C, AF647, DY654, and DL633, respectively, and the rest 22.9% events were rejected because of ambiguity. (Fig. 4j and Supplementary Table 1).

### 3D tetra-color ExR-STORM imaging of COS-7 cells

Cell organelles and their interaction sites are distributed in 3D, so axial resolving capability is important for multicolor imaging. We achieved 3D tetra-color imaging by combining the astigmatic method with the ExR-STORM system. In 3D ExR-STORM, the resonant mirror introduced negligible influence on the point spread function (PSF), and the fitting process was compatible with the elliptical PSF (Supplementary Fig. S5). The localization precision was approximately 8 and 23 nm in the lateral and axial directions, respectively (Supplementary Fig. S4), which is consistent with previous single-color results^24^.

**Fig. 5:**
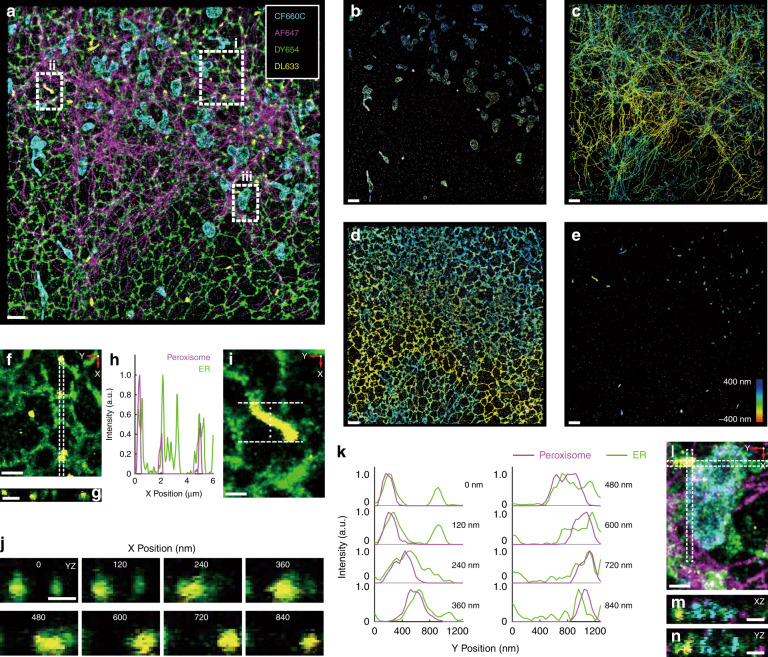
3D Tetra-color imaging based on ExR-STORM. **a-e** CF660C, Alexa Fluor 647, Dyomics 654, and DyLight 633 were selected to label the outer mitochondrial membrane (cyan), intermediate filaments (magenta), endoplasmic reticulum (green), and peroxisomes (yellow), respectively, in a fixed COS-7 cell. **a** Reconstructed image of the four merged channels. **b**–**e** Color-coded four channels in (**a**). **f**–**k** Colocalization of peroxisomes and endoplasmic reticulum. **f** Zoomed-in view of boxed region **i** in (**a**). **g** XZ (120 nm in Y) cross-section of the boxed region in (**f**). **h** Intensity profile along the boxed region in (**f**). **i** Zoomed-in view of the boxed region **ii** in (**a**). **j** YZ (120 nm in X) cross-sections of the region between the two dashed lines in (**i**), where the distance between adjacent cross-sections in the X direction is 120 nm. **k** Intensity profiles along the Y direction in (**i**), with each profile corresponding to the cross-section image in (**j**). **l** The ER–mitochondria–peroxisome tripartite contact site in the boxed region **iii** in (**a**). **m**, **n** XZ (120 nm in Y) and YZ (120 nm in X) cross-sections of the boxed regions in (**l**). Scale bars: 2 μm (**a**–**e**); 1 μm (**f**–**g**); 500 nm (**i**, **j, l**–**n**)

ER exhibits rich interactions with other organelles^15,27^. Therefore, ER, intermediate filaments, peroxisome, and the outer mitochondrial membrane were labeled in fixed COS-7 cells (Fig. 5a–e and Supplementary Video 2) for interaction analysis. We found that peroxisomes colocalized with ER in the XY, YZ, and XZ cross-sections (Fig. 5f–k), elucidating the relationships between peroxisomes and ER^27,28^. Additionally, a mitochondrion contacted peroxisome, and both of them colocalized with the ER (Fig. 5l–n), indicating the existence of tripartite contact sites between peroxisomes, ER, and mitochondria^29,30^. Colocalization between ER and intermediate filaments was also observed in Fig. 5a.

Mitochondrial double-stranded DNA (dsDNA), intermediate filaments, and the outer mitochondrial membrane were also imaged with 3D ExR-STORM (Supplementary Fig. S6 and Supplementary Video 3). Cross-sections helped to reveal the relative 3D position of diffraction-limited structures–for example, the hollow structures of the outer mitochondrial membrane (Supplementary Fig. S6b–d), the dsDNA inside the mitochondria (Supplementary Fig. S6b–d) and intermediate filaments in contact with the mitochondria (Supplementary Fig. S6c, d).

## Discussion

In this work, we combined multi-wavelength excitation with a fast switching scheme and successfully resolved four spectrally very close far-red dyes (DyLight 633, Alexa Fluor 647, Dyomics 654, and CF660C) simultaneously with negligible cross-talk and little chromatic aberrations.

Compared with other multicolor schemes in SMLM, our proposed method has several advantages. First, dyes with similar emission spectra could be resolved with ExR-STORM, resulting in negligible chromatic aberrations and providing more choices of dyes. In this study, we successfully resolved Dyomics 654 and Alexa Fluor 647, which have emission peaks that are only 5 nm apart. Second, ExR-STORM resolved four far-red dyes with only one objective, extending color channels with cross-talk of less than 3%. Third, differing from other sequential imaging with multi-wavelength excitation methods^19–21^, which would suffer from the blinking effects of STORM dyes, we used a 4 kHz scanner (250 μs per imaging cycle) to eliminate the brightness fluctuation, compatible with fast dynamics of single molecule dyes. Fourth, since the PSF of ExR-STORM occupied fewer pixels than the spectrum in spectrally resolved STORM did^17,31^, a higher molecule density could be realized, resulting in higher molecular throughput.

In our tetra-color experiment, the molecule density was 0.2–0.3 points/μm^2^. In this condition, most of the single molecules were not overlapped. We estimated the relationship between the molecule density and cross-talk ratio through simulations and found that the cross-talk ratio and the rejection ratio increased with increasing molecule density (Supplementary Fig. S7). Therefore, it is necessary to balance molecule density to a certain level where no evident cross-talk is introduced.

ExR-STORM successfully achieved tetra-color superresolution imaging, and more color channels could be extended by adopting the following two approaches. First, additional excitation lasers could be used to obtain more channels, which will also contribute to less cross-talk. Second, ExR-STORM could be combined with existing emission spectra-based multicolor schemes^12–16^, such as introducing a dichroic mirror before the resonant mirror. With these two modifications, more colors could be used in the palette of multicolor SMLM in the future.

Various cell organelles regularly interact with each other through direct membrane contact. Our ExR-STORM system provides a new approach to studying these interactions, and several organelle contacts and colocalization were observed in three-dimension in this work. We believe that ExR-STORM will allow further investigation of organelle organization and interactions in cell stress responses, migration, division, and differentiation.

## Materials and methods

### Optical setup

The optical setup was built based on an Olympus IX73 inverted microscope (Supplementary Fig. S1). Lasers with wavelengths of 620 nm (1 W, MPB Communications), 639 nm (1 W, CNI), and 671 nm (1 W, CNI) were coupled to an optical fiber after being modulated by the AOTF and then focused on the back focal plane of the objective (100×, 1.50 N.A., Olympus) to generate total internal reflection fluorescence illumination (TIRF). A dichroic mirror (FLD 681 DLP, Iridian) was used to reflect the excitation light and transmit the emission light. Emission filters (BLP01-664R, Semrock; ET740sp, Chroma) were installed to filter the excitation laser and infrared noise.

In the imaging path, a field diaphragm (GCM5711M, Daheng Optics) was placed at the first image plane to control the field of view (FOV) of the three subregions. A linear slide (GCM-TD50MX, Daheng Optics) was used for switching the cylindrical lenses (*f* = 500 mm, LJ1144L1-A, Thorlabs) into imaging paths, changing the image mode between 2D and 3D. The fluorescence images were then focused on a 4 kHz resonant scanning mirror (CRS 4 KHZ, CTI) after passing through 2 lenses (*f* = 180 mm, AC508-180-A-ML, Thorlabs; *f* = 150 mm, AC508-150-A-ML, Thorlabs). Then, the fluorescence images were reflected by the scanner to three light paths, each with a pair of lenses, generating three subimages on the EMCCD (iXon 897 EMCCD, Andor, Supplementary Fig. S3). Because the resonant mirror was placed at the image plane, the final image on the CCD was not blurred when the resonant mirror was scanning (Supplementary Fig. S8). During imaging, the excitation lasers were turned off when the fluorescence beam was located at the junction between the two lenses to avoid signal loss and cross-talk. During image acquisition, the resonant mirror generated a 4 kHz signal, then multiplied by a complex programmable logic device, and the output signal with a frequency of ~800 kHz was taken as a clock source for the data acquisition device (DAQ, NI USB-6343, National Instruments) to modulate the AOTF^22,23^. The details of the resonant scan sequence diagram and the image path-switching process are shown in Supplementary Fig. S2.

### Validation of the co-fitting method

To validate the performance of the co-fitting method, one hundred groups of microsphere image stacks (each with ten frames) were obtained. The recorded raw data were first split and transformed to create three aligned subimage stacks. Then, three-channel co-fitting and independent fitting (fitting with one channel) were conducted. Points that persisted for ten frames were extracted, and the positions of the four fitting results were aligned by subtracting the center of the points in the co-fitting results. Then, statistical analyses of the registration errors of all groups were performed.

### ExR-STORM imaging

The whole image is 512 × 512 pixels, and the three subimages were acquired in three different subregions of the EMCCD. The size of each subregion is approximately 220 × 220 pixels, with a pixel size of 160 nm, yielding a FOV of approximately 35 × 35 μm^2^.

For cellular imaging, the average excitation power densities (620, 639, and 671 nm) were 1100, 800, and 1100 W/cm^2^, respectively. A 405 nm laser was used for density control of single molecules during image acquisition. The frame rate of the EMCCD was 20 Hz, the EM gain factor was 100, and the ADU conversion factor was 4.96. A total of 20,000–30,000 frames were acquired for image reconstruction, and molecules with lifetimes exceeding 5 frames or photon numbers of less than 300 were rejected, as weak signals were more influenced by background noise, thus introducing more uncertainty into molecule identification.

In 3D imaging, a cylindrical lens was introduced, and the relative axial position was calculated according to the calibration results of 0.1 μm TetraSpeck microspheres before cellular imaging (Supplementary Fig. S5). The calibration curve was acquired by fitting the astigmatic PSF, enabling the 3D position to be determined in the axial range of ±400 nm. In the axial direction, the resonant mirror introduced negligible error to the PSF (Supplementary Fig. S5b). The difference of PSF widths in the *x*-direction and *y*-direction changed monotonically with the z-position during scanning so that the axial position could be determined according to a three-order polynomial fitting curve (Supplementary Fig. S5c).

The oxygen-depleted STORM imaging buffer consisted of 10% glucose, 1× phosphate-buffered saline (PBS) buffer, GLOX (catalase (0.06 mg/mL) and glucose oxidase (0.6 mg/mL), dissolved in Tris-HCl buffer) and 143 mM 2-mercaptoethanol.

### Calibration and drift correction

The 0.1 μm TetraSpeck microspheres (T7279, Invitrogen) were used for image registration before cellular imaging. Image stacks of the bright microspheres were acquired and combined to increase the signal-to-noise ratio (SNR); and then, image splitting and registration were performed to obtain the transform matrix. All subimages were aligned to the 639 nm light path.

To ensure that the sample remained in the focal plane during image acquisition, a TIRF Lock system was used for real-time axial drift correction with PIEZO (P-725.4CD, PI), maintaining the focal plane according to the feedback from the center of the exiting TIRF beam collected by a CCD. To obtain the feedback coefficient for real-time focus locking, we scanned ±600 nm of the focal plane in the step of 10 nm and used least squares regression to fit the axial position and the center position of the exiting TIRF beam. The drift of the focal plane was kept within ±20 nm during image acquisition.

Redundant cross-correlation (RCC)^32^ was used for lateral drift correction during postprocessing. Five hundred frames were set for the correlation steps according to the structure distribution and point density.

### Sample preparation

#### Cell culture

COS-7 cells (3111C0001CCC000033, National Infrastructure of Cell Line Resources, China) were grown in Dulbecco’s modified Eagle medium (DMEM) (C11995500BT, Gibco) supplemented with 10% fetal bovine serum (16000–044, Gibco) and 100 U/mL penicillin–streptomycin solution (SV30010, HyClone) at 37 °C with 5% CO_2_.

#### Nanobody and antibody conjugation

Nanobody against GFP (gt-250, Chromotek) was conjugated to Dyomics 654-NHS Ester (654-01, Dyomics). The conjugation reaction was carried out in 0.1 M sodium bicarbonate in the dark for 1 h. Then we used a Zeba Spin Desalting Column (89882, Thermo Scientific) with a molecular weight cutoff of 7 kDa to remove excess dye from the conjugation reaction. Nanobodies were incubated at 1:50 dilution at room temperature for 1 h.

Unlabeled donkey anti-mouse IgG (ab6707, Abcam) was conjugated with Dyomics 678-NHS Ester (678-01, Dyomics), Dyomics 675-NHS Ester (675-01, Dyomics) or Alexa Fluor 660-NHS Ester (A20007, Invitrogen). The reaction was carried out in 0.1 M sodium bicarbonate in the dark for 1 h. Excess dye was removed from the antibody using a Pro-Spin Column (CS800, Princeton Separations) according to the manufacturer’s recommendations.

#### Sample labeling

##### Microtubule samples

To label the microtubules, COS-7 cells with a density of 50,000 were cultured in 35 mm glass-bottomed dishes (D35-14-1-N, Cellvis). Twenty-four hours later, the cells were rinsed with PBS (P4417, Sigma-Aldrich). Then, the cells were immobilized with 0.1% glutaraldehyde (16220, electron Microscopy Science) and 3% paraformaldehyde (157-8, electron Microscopy Science) in PBS at room temperature for 10 min. The reduction process was conducted in PBS with 0.1% sodium borohydride (71320, Sigma-Aldrich) for 7 min. Following washing with PBS, cell permeation was performed with 0.2% Triton X-100 (93443, Sigma-Aldrich) in PBS for 7 min. Samples were blocked with 10% NGS (C0265, Beyotime) and 0.05% Triton X-100 in PBS at room temperature for 90 min, then incubated with primary antibodies in antibody dilution buffer (0.05% Triton X-100, 5% NGS in PBS) for 60 min. The cells were then washed 5 times with washing buffer (0.05% Triton X-100, 1% NGS in PBS), each time for 15 min, and then stained for 60 min with secondary antibodies. Next, the cells were washed with washing buffer 5 times and fixed with 4% paraformaldehyde in PBS for 10 min. Then, the cells were washed with PBS 3 times, and ddH_2_O for 3 times, both 5 min per wash. At last, the samples in ddH_2_O were stored at 4 °C. The primary antibody used was mouse anti-α-tubulin (T9026, Sigma–Aldrich, used at 1:100 dilution). Secondary antibodies labeled with Alexa Fluor 647 (A31571, Invitrogen, used at 1:100 dilution), DyLight 633 (35512, Invitrogen, used at 1:300 dilution), and Dyomics 678, Dyomics 675, and Alexa Fluor 660 (conjugated in the laboratory; see *Nanobody and antibody conjugation* section; used at 1:50, 1:50, and 1:100 dilutions, respectively) were used to label primary antibodies.

##### ER samples

Lentiviral particles of GFP-Sec61β were packaged by OBiO Technology (Shanghai) according to the authors’ designs and requirements. The titer of the lentivirus used in the experiments was 5.4 × 10^8^ infectious units/mL. COS-7 cells were infected with lentivirus according to the manufacturer’s protocols provided by OBiO Technology. After 48 h, the infected cells with a density of 50,000 were cultured in 35 mm glass-bottomed dishes. The following immunofluorescence steps were performed as described in the Microtubule samples section. Nanobody GFP-binding protein conjugated with Dyomics 654 (see Nanobody and antibody conjugation section; used at 1:50 dilution) was used to label GFP-Sec61β.

##### Mitochondria samples

To label mitochondria, COS-7 cells with a density of 50,000 were cultured in 35 mm glass-bottomed dishes. After 24 h, immunofluorescence was performed as described in the Microtubule samples section. The primary antibody used was rabbit anti-TOMM20 (ab186735, Abcam, used at 1:250 dilution). Secondary antibodies labeled with CF660C (20815, Biotium, used at 1:100 dilution) and CF680 (20820, Biotium, used at 1:500 dilution) were used to label primary antibodies.

##### Intermediate filament samples

To label intermediate filament samples, COS-7 cells with a density of 50,000 were cultured in 35 mm glass-bottomed dishes. After 24 h, immunofluorescence was performed as described in the Microtubule samples section. The primary antibody used was chicken anti-vimentin (ab24525, Abcam, used at 1:200 dilution). Secondary antibodies labeled with Alexa Fluor 647 (A21449, Invitrogen, used at 1:150 dilution) were used to label primary antibodies.

For the mitochondria/dsDNA/intermediate filament three-color sample, COS-7 cells with a density of 50,000 were cultured in 35 mm glass-bottomed dishes. After 24 h, immunofluorescence was performed as described in the Microtubule samples section. The rabbit anti-TOMM20, mouse anti-dsDNA (ab27156, Abcam), and chicken anti-vimentin dilution ratios were 1:250, 1:2000, and 1:200, respectively. Secondary antibodies of the three-color sample were labeled with CF660C (1:100)/DyLight 633 (1:2 000)/Alexa Fluor 647 (1:150).

For the ER/microtubule/mitochondria/intermediate filament four-color sample, COS-7 cells were infected with lentiviral particles of GFP-Sec61β. The infected cells with a density of 50,000 were cultured in 35 mm glass-bottomed dishes, and immunofluorescence was performed as described in the Microtubule samples section. The mouse anti-α-tubulin, rabbit anti-TOMM20, and chicken anti-vimentin dilution ratios were 1:400, 1:250, and 1:200, respectively. Secondary antibodies of the four-color sample were labeled with Dyomics 654 (1:50)/DyLight 633 (1:400)/CF660C (1:100)/Alexa Fluor 647 (1:150).

For the ER/peroxisome/mitochondria/intermediate filament four-color sample, COS-7 cells were infected with lentiviral particles of GFP-Sec61β. The infected cells with a density of 50,000 were cultured in 35 mm glass-bottomed dishes, and immunofluorescence was performed as described in the Microtubule samples section. The mouse anti-PMP70 (SAB4200181, Sigma-Aldrich), rabbit anti-TOMM20, and chicken anti-vimentin dilution ratios were 1:100, 1:250 and 1:200, respectively. Secondary antibodies of the four-color sample were labeled with Dyomics 654 (1:50)/DyLight 633 (1:50)/CF660C (1:100)/Alexa Fluor 647 (1:150).

## Supplementary information

Supplementary Material: Tetra-color superresolution microscopy based on excitation spectral demixing

supplement video 1

supplement video 2

supplement video 3

## Data Availability

The data that support the findings of this study are available from the corresponding author upon request.
