# Supplementary material for: Tetra-color superresolution microscopy based on excitation spectral demixing
Source: Light Sci Appl. 2023 Jan 2;12:9. doi: 10.1038/s41377-022-01054-6 (PMC9806106; doi:10.1038/s41377-022-01054-6)

**Supplementary Material:**

**Tetra-color superresolution microscopy based on excitation spectral demixing**

Wanyan Wu^1,2,#^, Shihang Luo^1,2,#^, Chunyan Fan^3,#^, Tianjie Yang^1,4^, Shuwen Zhang^1^,

Wenxiang Meng^3,5^, Tao Xu^1,2,4,6,7,*^, Wei Ji^1,2,6,*^, Lusheng Gu^1,2,6,*^

^1^ Institute of Biophysics, Chinese Academy of Sciences, Beijing, 100101, China.

^2^ College of Life Science, University of Chinese Academy of Sciences, Beijing, 100049, China.

^3^ State Key Laboratory of Molecular Developmental Biology, Institute of Genetics and Developmental Biology, Innovation Academy for Seed Design, Chinese Academy of Sciences, Beijing, 100101, China.

^4^ School of Future Technology, University of Chinese Academy of Sciences, Beijing, 100049, China.

^5^ College of Advanced Agricultural Sciences, University of Chinese Academy of Sciences, Beijing, 100049, China.

^6^ Bioland Laboratory, Guangzhou, 510005, China.

^7^ Guangzhou Laboratory, Guangzhou, 510030, China.

^#^ These authors contributed equally: Wanyan Wu, Shihang Luo, Chunyan Fan.

* Corresponding author. Email: [xutao@ibp.ac.cn](mailto:xutao@ibp.ac.cn) (T.X.); [jiwei@ibp.ac.cn](mailto:jiwei@ibp.ac.cn) (W.J.); [gulusheng@ibp.ac.cn](mailto:gulusheng@ibp.ac.cn) (L.G.).

**Index**

[Supplementary Figure 1: Optical setup 3](#_Toc120629058)

[Supplementary Figure 2: Time sequence diagram 4](#_Toc120629059)

[Supplementary Figure 3: One frame of raw images from the EMCCD 5](#_Toc120629060)

[Supplementary Figure 4: Three-dimensional localization distribution for single molecules of different dyes 6](#_Toc120629061)

[Supplementary Figure 5: Axial position calibration 7](#_Toc120629062)

[Supplementary Figure 6: 3D ExR-STORM images of dsDNA, intermediate filaments and the outer mitochondrial membrane 8](#_Toc120629063)

[Supplementary Figure 7: The simulation of cross-talk and rejection ratio under different molecule densities 10](#_Toc120629064)

[Supplementary Figure 8: Comparison of the PSF width when the resonant mirror was on and off 11](#_Toc120629065)

[Supplementary Video 1: Raw camera images 12](#_Toc120629066)

[Supplementary Video 2: Four-color reconstructed image 12](#_Toc120629067)

[Supplementary Video 3: Three-color reconstructed image 12](#_Toc120629068)

[Supplementary Table 1: Cross-talk and rejected fraction for the four-color imaging of CF660C, AF647, DY654 and DL633 in Fig. 4j. 13](#_Toc120629069)

# Supplementary Figure 1: Optical setup


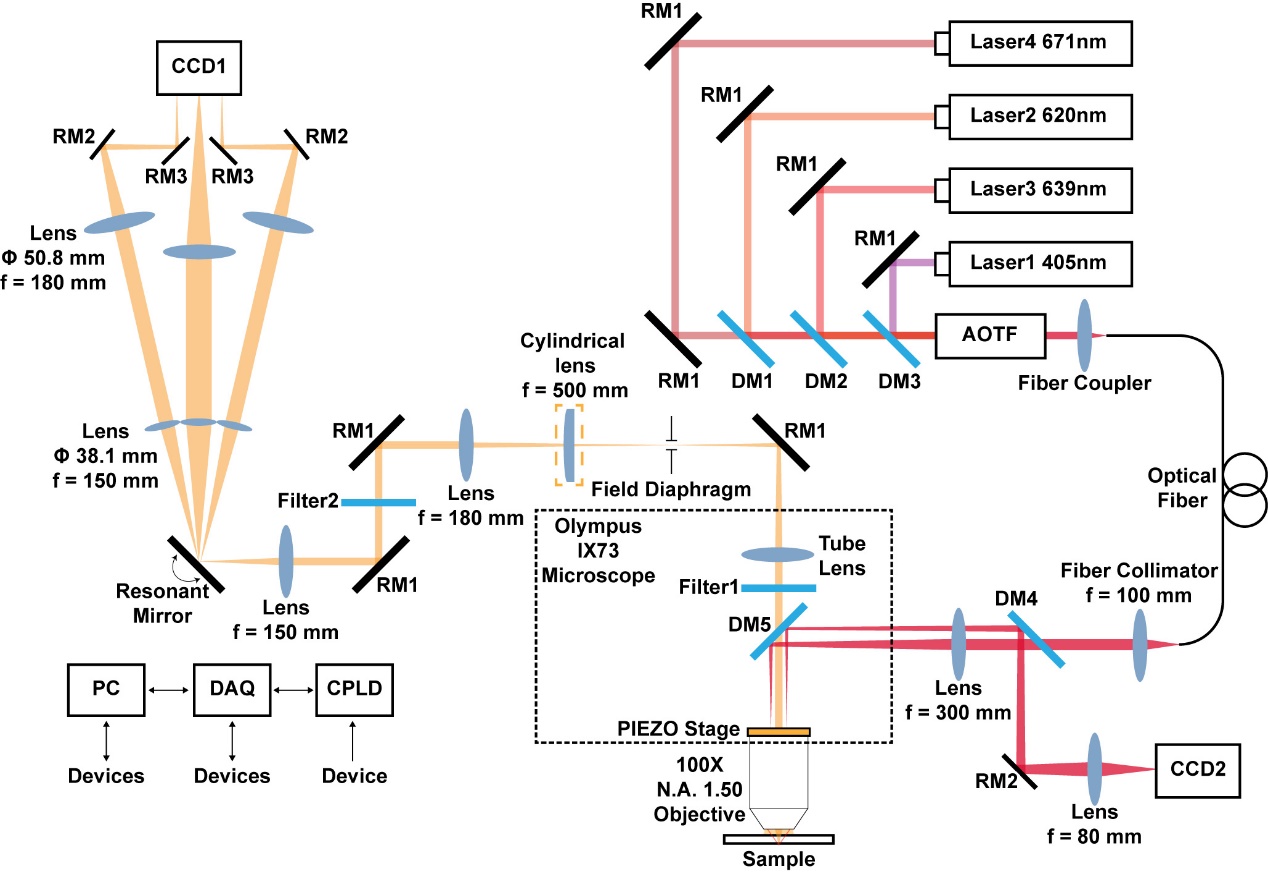


**Fig. S1. | Optical setup.** Laser1: WCP405-150FS, OBIS; Laser2: 2RU-VFL-P-1000-620-B1R, MPB; Laser3: MLL-FN-639, CNI; Laser4: MLL-FN-671, CNI; RM1: GCCH-101102, Daheng Optics; DM1: ZT640rdc-UF3, Chroma; DM2: ZT405/488/532/640rpc-XT, Chroma; DM3: ZT405/488/561rpc, Chroma; DM4: ZT488dcrb, Chroma; DM5: FLD 681 DLP, Iridian; AOTF: AOTFnC-VIS-TN, AA; Fiber Coupler: HPUC-2A3AHPC-400/700-S-6.3AC-11, OZ; Fiber: QSMJ-A3AHPCA3HPC-400/680-3/125-3AS-2, OZ; Fiber Collimator: SM1FC, Thorlabs; PIEZO Stage: P-725.4CD, PI; Filter1: BLP01-664R, Semrock; Filter2: ET740sp, Chroma; Resonant Mirror: CRS 4KHZ, CTI; RM2: BBSQ1-E02, Thorlabs; CCD1: iXon 897 EMCCD, Andor; CCD2: Guppy F-033B, Allied Vision Technologies.

# Supplementary Figure 2: Time sequence diagram


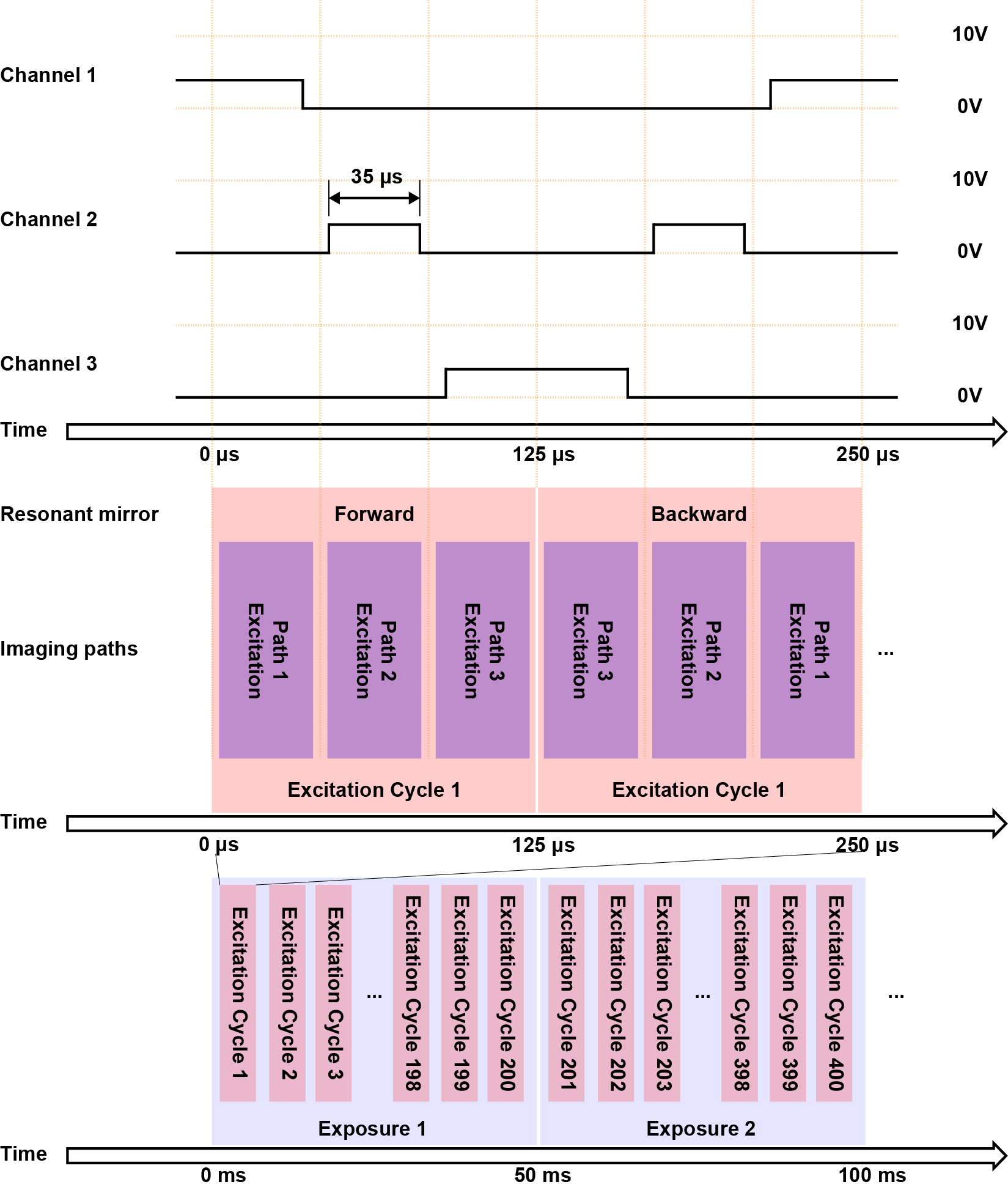


**Fig. S2. | Time sequence diagram.** This figure shows the signal that instructs the AOTF to switch the illumination patterns. The signal was generated by DAQ and synchronized with the resonant scanner. Each excitation cycle contains 6 illumination switch patterns, with the AOTF on-time of 35 μs × 6 = 210 μs, resulting in a duty cycle of approximately 84% for the illumination. Two exposure cycles of 50 ms are illustrated here, each with 200 excitation cycles.

# Supplementary Figure 3: One frame of raw images from the EMCCD


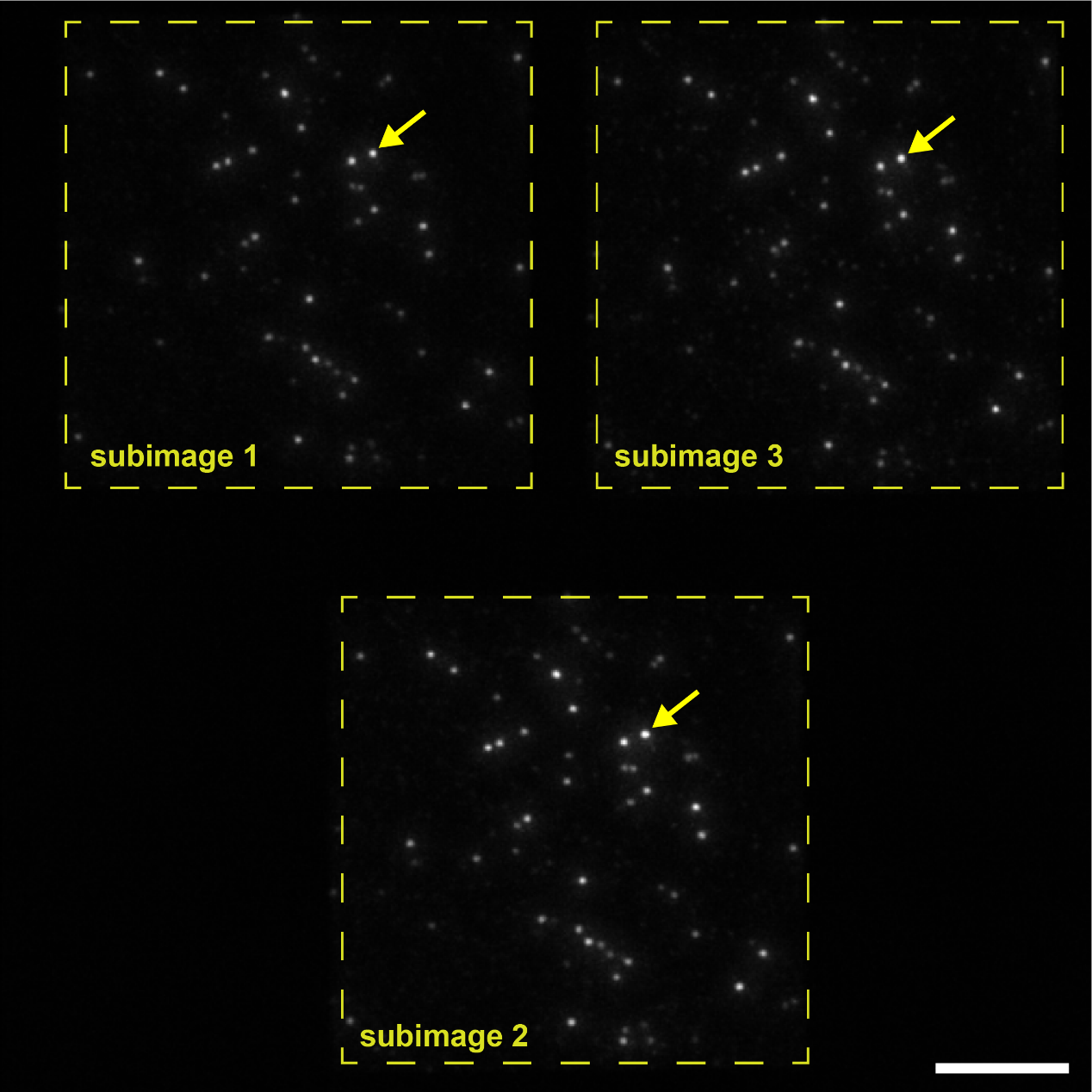


**Fig. S3. | One frame of raw images from the EMCCD.** The whole image is 512 × 512 pixels, and the three subimages are labeled as subimages 1-3. The size of each subimage is approximately 220 × 220 pixels, with a pixel size of 160 nm, yielding an FOV of approximately 35 × 35 μm^2^. The yellow arrows indicate the same point in the three subimages. Scale bars: 10 μm. Five experiments were repeated independently with similar results.

# Supplementary Figure 4: Three-dimensional localization distribution for single molecules of different dyes


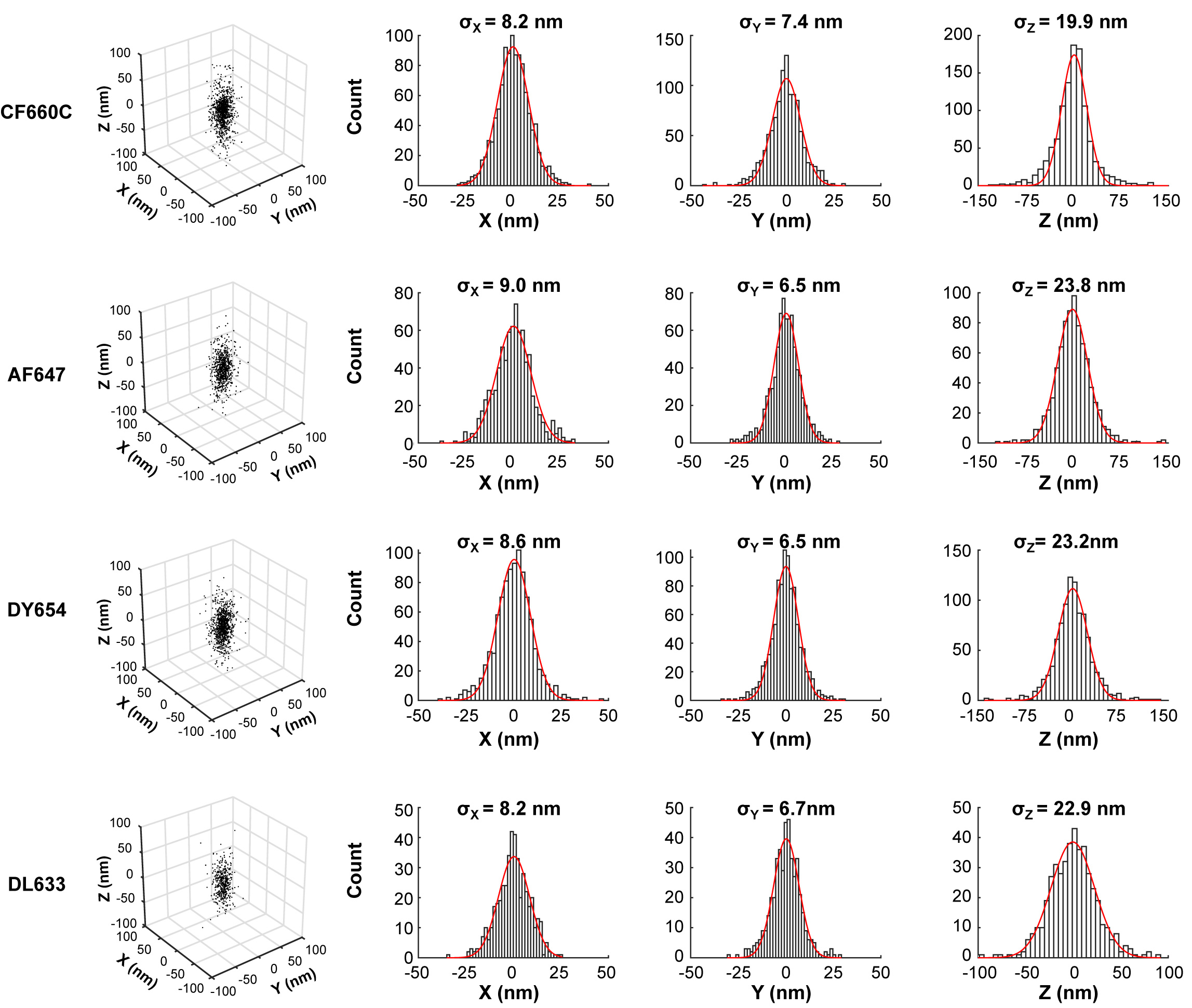


**Fig. S4. | Three-dimensional localization distribution for single molecules of different dyes.** Clusters of localizations were aligned according to their center-of-mass to generate the overall 3D presentation of the localization distribution. Localizations from >100 clusters (each with a molecule lifetime greater than seven continuous frames) were summarized for each dye channel. Histograms of the distribution in the x, y and z directions were fit to a Gaussian function, yielding standard deviations of ~ 8 nm in the lateral direction and of ~23 nm in the axial direction.

# Supplementary Figure 5: Axial position calibration

**
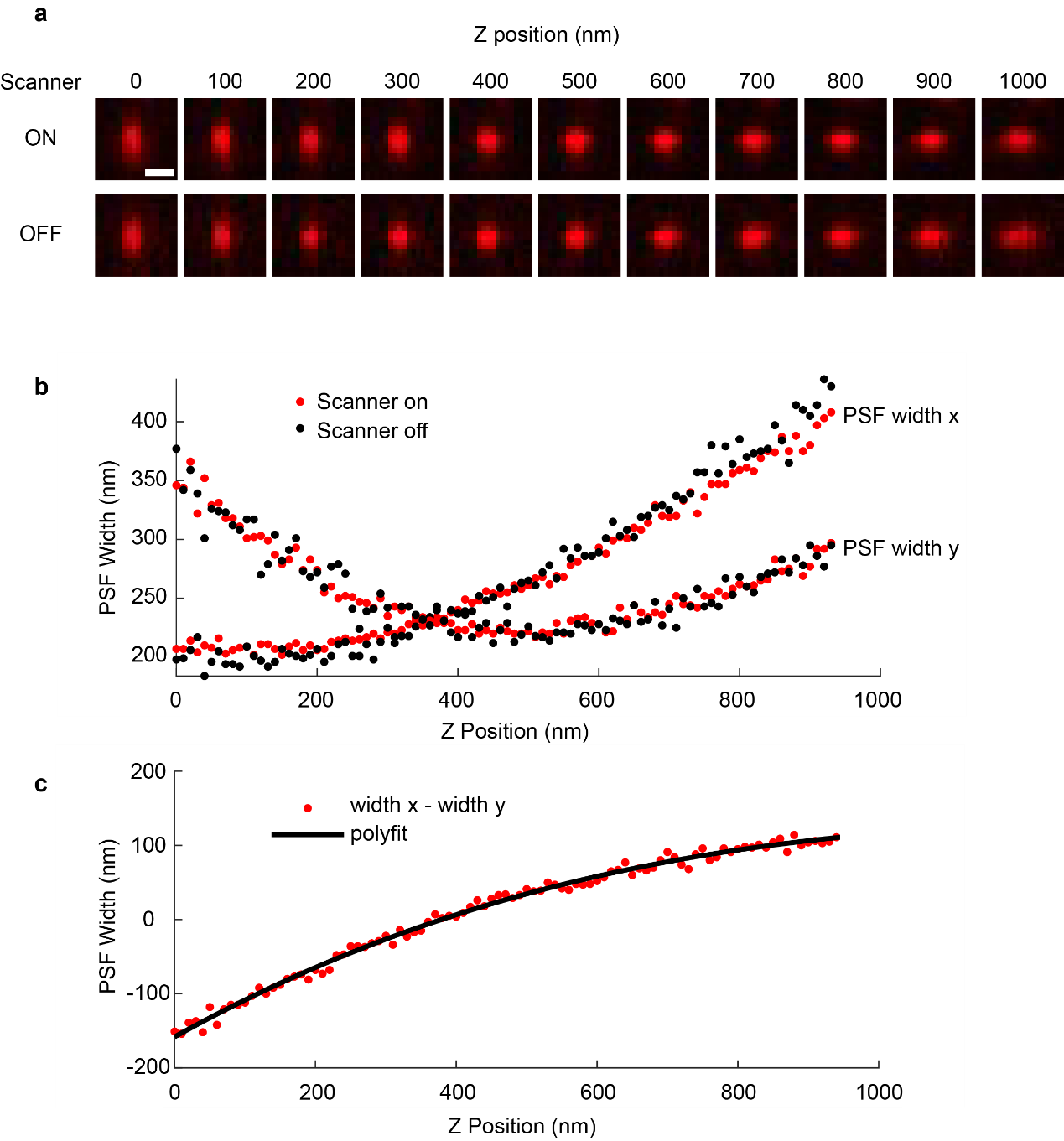
**

**Fig. S5. | Axial position calibration.** (a) Images of one fluorescent microsphere at different axial positions within the depth of field when the scanner was on and off. (b) The centroid fitting results show similar widths in the x and y directions, indicating that the scanning mirror introduces negligible image blur to the whole depth of field. (c) The width difference shows a monotonic relationship with the axial location when the scanner is working, and a 3-order polynomial fitting curve is used to calculate the axial position. Scale bars: 1 μm (a). For the data in (a) and (b), 5 experiments were repeated independently with similar results.

# Supplementary Figure 6: 3D ExR-STORM images of dsDNA, intermediate filaments and the outer mitochondrial membrane


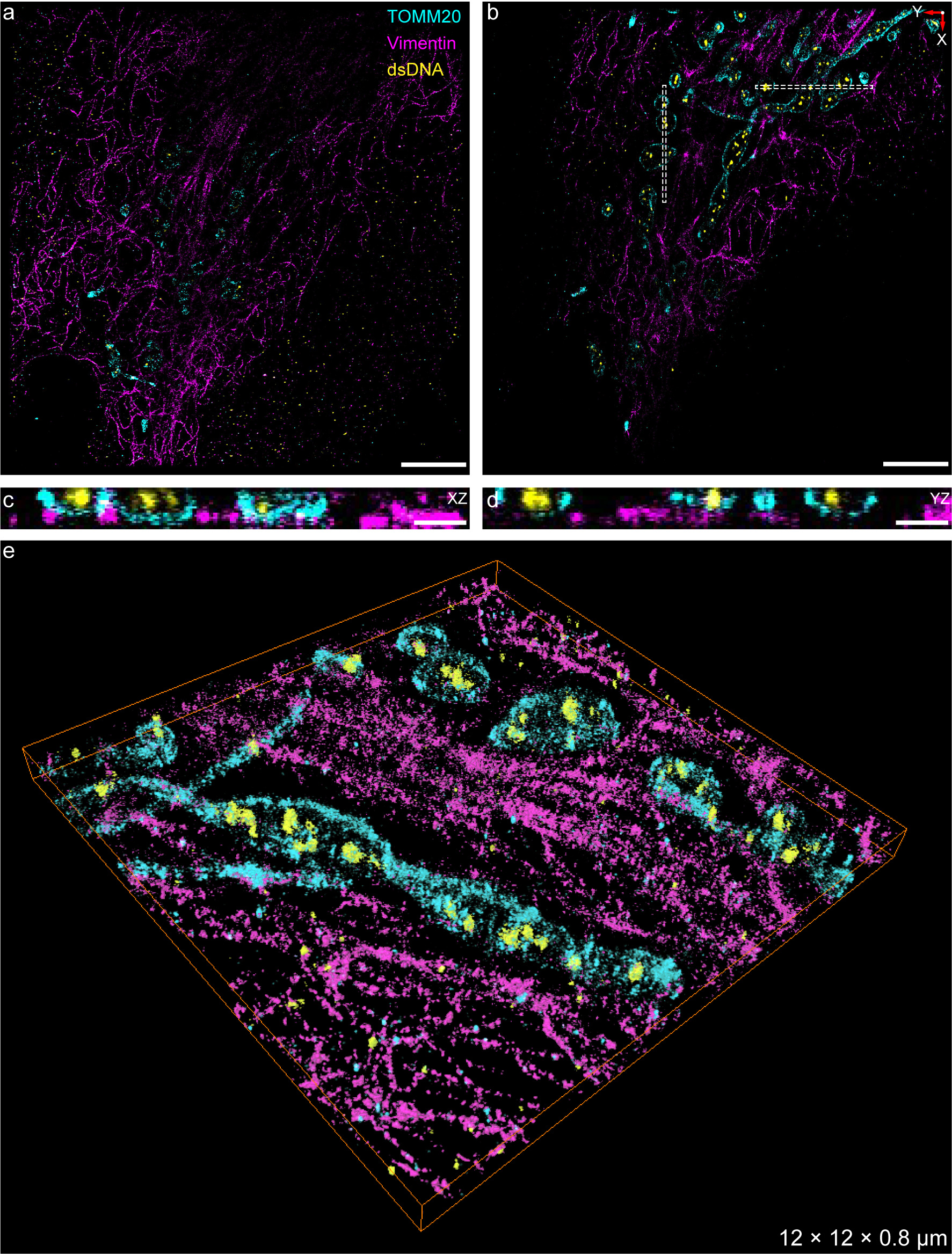


**Fig. S6. | 3D ExR-STORM images of mitochondrial dsDNA, intermediate filaments and the outer mitochondrial membrane.** CF660C, Alexa Fluor 647 and DyLight 633 were selected to label the outer mitochondrial membrane, intermediate filaments and mitochondrial dsDNA in a fixed COS-7 cell, respectively. (a-b) XY (120 nm in Z) cross-sections of the upper (a) and middle (b) layers of the mitochondria, respectively. (c-d) XZ (120 nm in Y) and YZ (120 nm in X) cross-sections of the boxed regions in (b). (e) Three-color reconstruction image in 3D. Scale bars: 5 μm (a-b), 1 μm (c-d).

# Supplementary Figure 7: The simulation of cross-talk and rejection ratio under different molecule densities


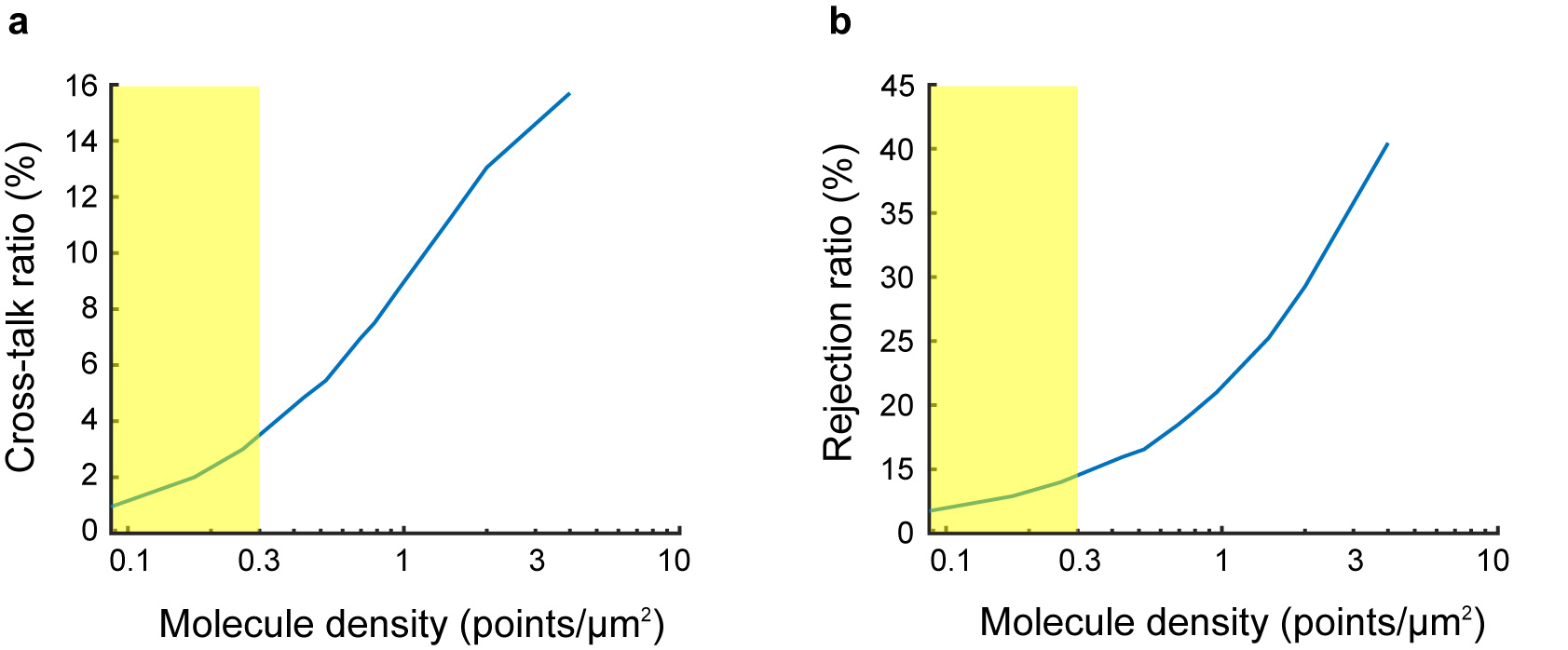


**Fig. S7. | The simulation of cross-talk and rejection ratio under different molecule densities.** Dual-color images were generated and analyzed based on the experimental data for Alexa Fluor 647 and CF660C, where the mean photon number was 2500, consistent with the experimental conditions. For each dye, the photon number ratios among the 620, 639 and 671 paths were determined according to the experimental data. (a) The cross-talk ratio under different molecule densities. (b) The rejection ratio under different molecule densities. The yellow region indicates the optimal imaging condition where most molecules do not overlap.

# Supplementary Figure 8: Comparison of the PSF width when the resonant mirror was on and off


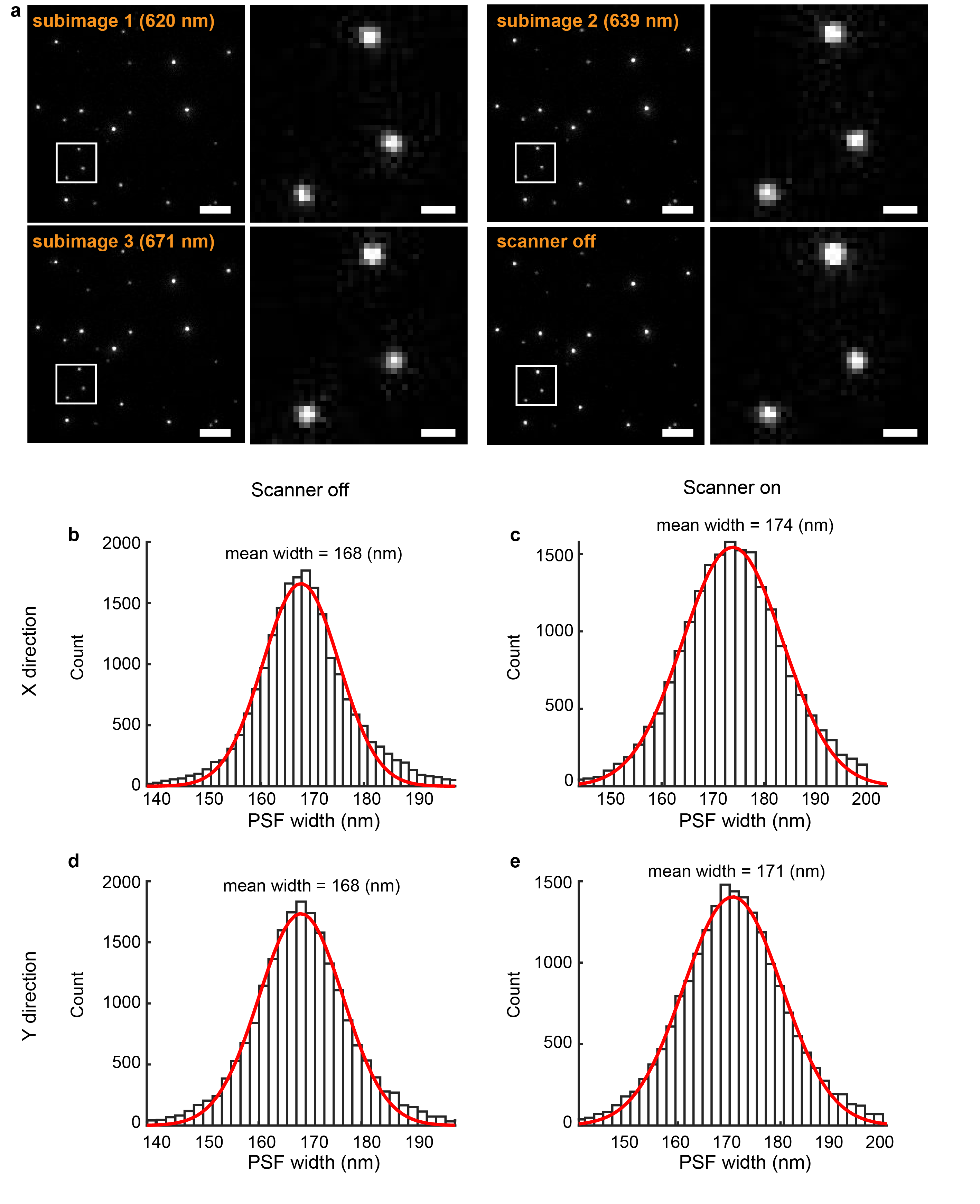


**Fig. S8. | Comparison of the PSF width when the resonant mirror was on and off.** (a) Three subimages of 100 nm fluorescent microspheres with and without scanning of the resonant mirror, with zoomed-in figures on the right indicated by the white box; Scale bars: 5 μm (left side of each image pair) and 1 μm (right side of each image pair). (b-e) Distributions of the PSF width (the standard derivation) of the 100 nm fluorescent microspheres in the x and y directions, with and without scanning of the resonant mirror. For the data in (a), 5 experiments were repeated independently with similar results.Additional Supplementary Material

## Supplementary Video 1: Raw camera images

Recorded single-molecule images of three light paths obtained within 5 s (100 frames) for the sample shown in Fig. 4a. The FOV was the same as that in Supplementary Fig. S3. Scale bar: 10 μm (MP4 940 KB).

## Supplementary Video 2: Four-color reconstructed image

Four-color reconstructed image of mitochondria (cyan), intermediate filaments (magenta), endoplasmic reticulum (green), and peroxisomes (yellow) for the sample shown in Fig. 5a. Scale bar: 5 μm (MP4 7435 KB).

## Supplementary Video 3: Three-color reconstructed image

Three-color reconstructed image of mitochondria (cyan), intermediate filaments (magenta) and dsDNA (yellow). Scale bar: 5 μm (MP4 6297 KB).

# Supplementary Table 1: Cross-talk and rejected fraction for the four-color imaging of CF660C, AF647, DY654 and DL633 in Fig. 4j.


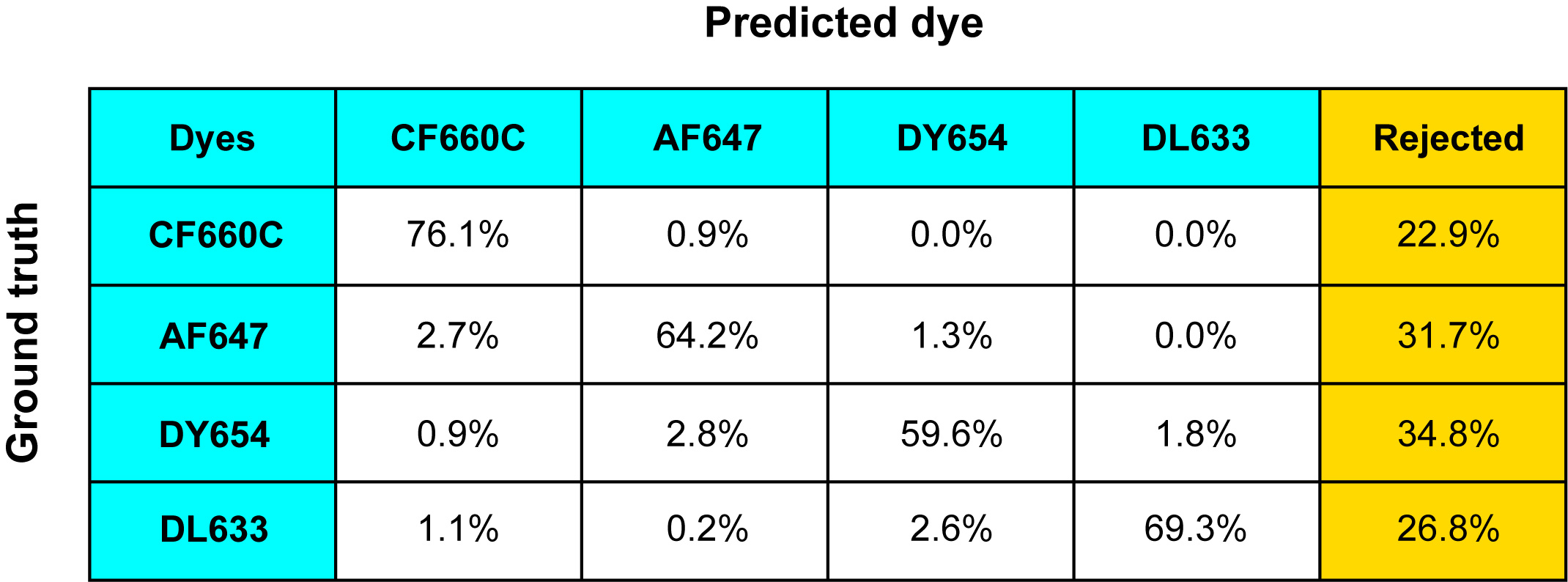

Supplement: Supplementary file 1 — Supplementary Material: Tetra-color superresolution microscopy based on excitation spectral demixing [file 41377_2022_1054_MOESM1_ESM.docx]
